# De novo ectopic hidradenitis suppurativa versus classic ectopic hidradenitis suppurativa: A case series and review of the literature

**DOI:** 10.1016/j.jdcr.2024.07.041

**Published:** 2024-08-30

**Authors:** Cassidy M. Nguyen, Pragna Naidoo, Venessa Peña-Robichaux

**Affiliations:** a Division of Dermatology, The University of Texas at Austin, Dell Medical School, Austin, Texas; b Medical Service, Section of Dermatology, Central Texas Veterans Healthcare System, Austin, Texas

**Keywords:** atypical, classic, de novo, ectopic, hidradenitis suppurativa

## Introduction

Hidradenitis suppurativa (HS) is a chronic follicular autoinflammatory disorder that presents with recurrent painful lesions, including nodules, abscesses, and tunnels, most commonly in intertriginous regions of the body. While initially believed to be a disease originating in the apocrine glands, current research points toward monogenic and polygenic mutations that predispose patients to follicular occlusion as well as auto-inflammatory and autoimmune processes that lead to characteristic recurring painful deep-seated nodules, draining abscesses, tunnel formation, and scarring.^1^

Pathologic confirmation is not necessary to establish diagnosis of HS as it is often diagnosed clinically. Three diagnostic criteria for HS have been proposed: the presence of typical lesions (eg, deep-seated painful nodules, abscesses, draining tunnels, bridged scars, double-ended comedones), occurrence of disease in one or more site of predilection (eg, axillae, inframammary, intermammary folds, groin, perineal region, buttocks), and the chronicity and recurrence of lesions.^2^

Although this diagnostic framework is applicable to most patients with HS, the evolving literature and our own patient experiences have continued to challenge the typical presentation of HS by describing multiple cases in which patients demonstrate HS lesions in nonintertriginous areas or in regions with a low density of apocrine glands.

HS lesions that appear in more uncommon areas are referred to as “ectopic HS.”^3^ However, within the ectopic cases reported, we have distinguished two groups of patients: (1) Those with ectopic HS who have a prior history of classic HS, which we refer to as “classic ectopic HS.” (2) Those who present with isolated ectopic lesions without a prior history of HS, which we refer to as “de novo ectopic HS.” We believe patients with de novo ectopic HS are likely clinically distinct from patients with classic ectopic HS and present two cases of patients with de novo ectopic HS. This is followed by a comparison of de novo and classic ectopic HS cases within the context of a retrospective review of the literature.

## Case 1

**Fig 1:**
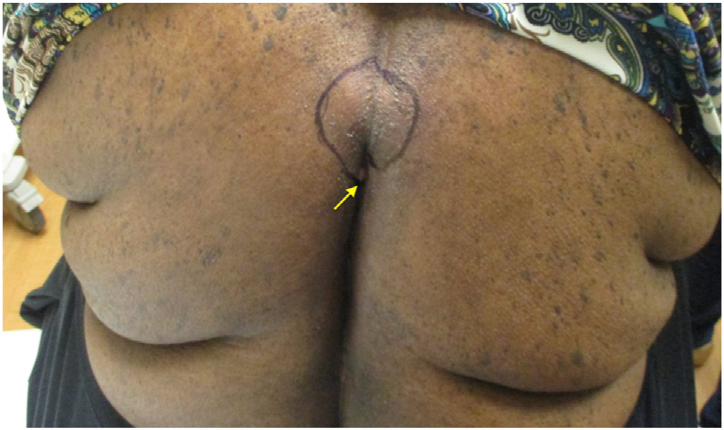
De novo ectopic hidradenitis suppurativa presenting as a chronic unremitting draining fistula at the *midline* of the upper back. The region *outlined* represents an erythematous area of induration and fluctuance with an ulcerated draining nodule at the inferior edge (*yellow arrow*).

A 68-year-old black female with a past medical history of obesity, diabetes, hypertension, hyperlipidemia, and a pilonidal cyst presented with a several month history of an unremitting abscess located on the mid-upper back that had been treated unsuccessfully with topical clindamycin 1% lotion, oral doxycycline 100 mg BID, and benzoyl peroxide 10% wash. She had no known prior history of HS but reported a previous abscess on her back located directly inferior to her current lesion that had since resolved. Physical examination revealed a 1.0-cm ulcerated nodule with hemorrhagic and purulent drainage at the midline of the upper back (Fig 1). Superior to this nodule was a 6.0 cm × 4.0 cm area of induration and central fluctuance with mild overlying erythema.

Given the lack of response to medical treatments, the chronic and recurrent nature of the lesion, as well as findings on clinical exam, a diagnosis of ectopic HS was made. It was believed that the ulcerated nodule represented the opening of a deep tunnel or fistula. The lesion was treated via surgical deroofing and left to heal by secondary intention. The patient was placed on doxycycline 100 mg twice daily for 4 weeks while the area healed. Histopathologic exam of tissue from the surgical procedure revealed abundant acute and chronic inflammation in the dermis, including chronic periadnexal inflammation, focal dermal granulation tissue, and fibrosis, which was suggestive of HS. No lining epithelium or keratin debris were identified, which made a ruptured epidermal cyst unlikely. The area was healed after six weeks, and the patient has not had any recurrence at that site to date.

## Case 2

**Fig 2:**
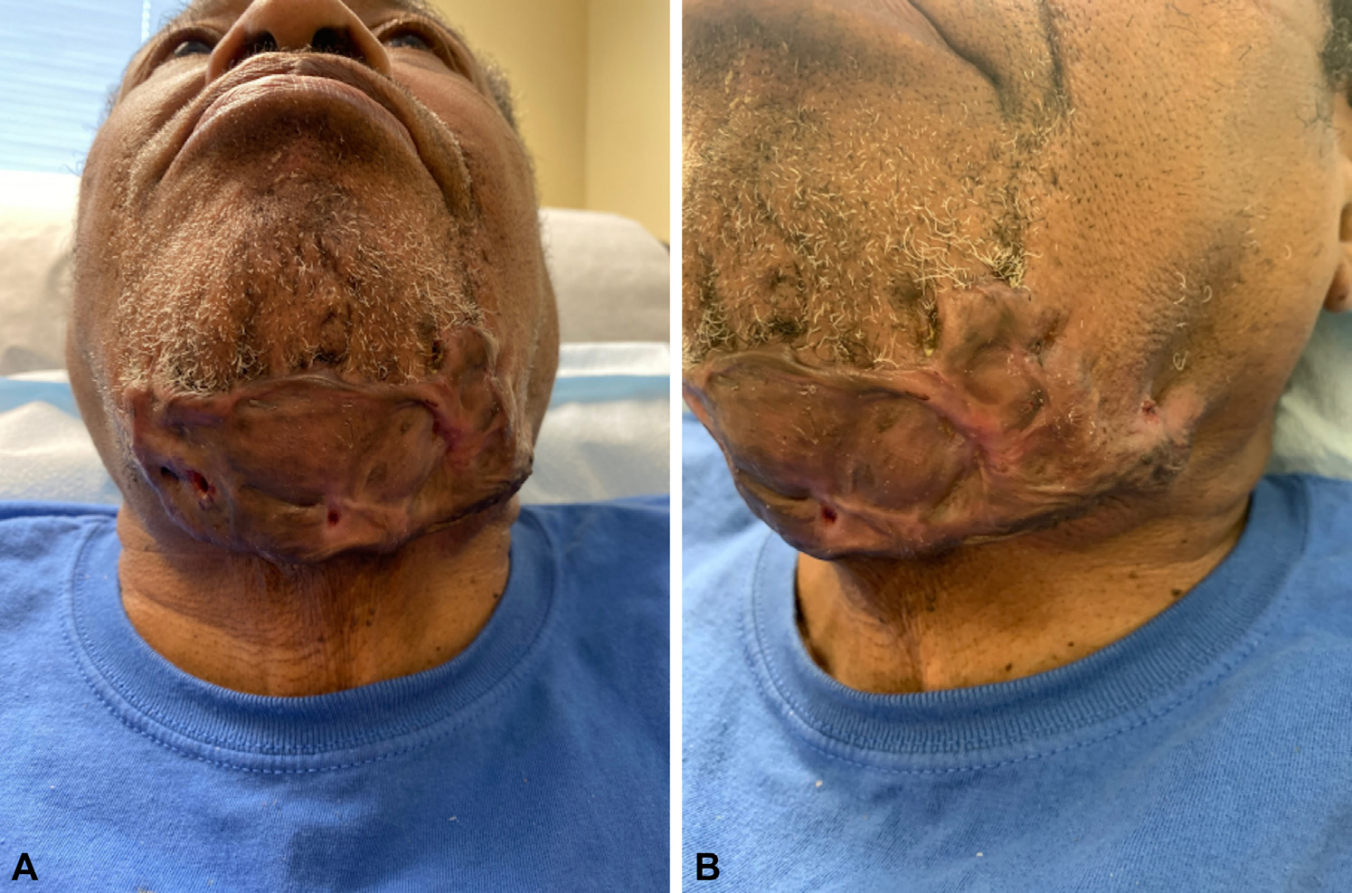
**A,** De novo ectopic hidradenitis suppurativa presenting as a chronic multiple interconnected draining tunnels within a keloidal scar in the submental region of the face. **B,** Closer image of lesion along the left jawline extending to the submental region.

A 74-year-old black male with a significant history of keloid scarring over the last 40 years presented with a draining, painful keloid scar of the submental region. He stated the lesion stemmed from a collection of ingrown hairs and slowly progressed to its current state over the last 10 to 12 years. Previous treatments included intralesional triamcinolone, oral clindamycin 300 mg BID, and oral doxycycline 100 mg BID. His physical exam was remarkable for a keloidal plaque along the submental area with underlying edema and multiple draining interconnected tunnels (Fig 2). He had no known history of HS.

A 4-mm punch biopsy of the lesion was sent for H&E, and a 6-mm punch biopsy was sent for fungal, bacterial, and atypical mycobacterial tissue cultures. Tissue cultures were negative and histopathologic findings yielded fibrosis and a mixed-cell inflammatory infiltrate suggestive of HS within a keloid scar. The patient was placed on 3 months of oral clindamycin 300 mg BID and rifampin 300 mg BID, after which he reported decreased pain and drainage of the facial lesion. After completion of the course of antibiotics, he started adalimumab 40 mg per week. He continued to report ongoing improvement with both decreased pain and drainage.

## Discussion

Although HS is classically described as a condition that presents with lesions in intertriginous areas, patients may also present with lesions in atypical areas known as ectopic HS. We have observed that patients with ectopic HS present either with ectopic lesions alone (de novo ectopic HS) such as our cases described, or in the setting of a classic presentation of HS (classic ectopic HS). Upon review of the literature, not including our cases presented, a total of 36 cases of ectopic HS have been described. We stratified these cases by de novo ectopic HS and classic ectopic HS presentation in order to provide deeper insight into potential trends and associated characteristics (Table I).

**Table I:** Summary of de novo ectopic HS and classic ectopic HS cases reported in the literature

	De novo ectopic^3^, ^4^, ^5^, ^6^, ^7^, ^8^, ^9^, ^10^, ^11^, ^12^, ^13^, ^14^	Classic ectopic^9^^,^^11^, ^12^, ^13^, ^14^, ^15^, ^16^, ^17^, ^18^, ^19^, ^20^, ^21^, ^22^, ^23^, ^24^, ^25^, ^26^, ^27^, ^28^, ^29^	Total
Cases (#)	12	24	36
Demographics			
Male (%, *n*)	67%, 8	67%, 16	67%, 24
Female (%, *n*)	33%, 4	33%, 8	33%, 12
Mean age (y)	44.6	38.5	40.5
Anatomical sites			
Head and neck (%, *n*)	25%, 3	54.2%, 13	44%, 16
Trunk (%, *n*)	33.3%, 4	25%, 6	27.8%, 10
Extremities (%, *n*)	33.3%, 4	33.3%, 8	33.3%, 12
Amputation stump (%, *n*)	16.7%, 2	8.30%, 2	11.1%, 4
Genitals (%, *n*)	0%, 0	8%, 3	8.3%, 3
C-section scar (%, *n*)	0%, 0	6%, 2	5.6%, 2
Comorbidities			
Diabetes (%, *n*)	42%, 5	8.3%, 2	19.5%, 7
Obesity (%, *n*)	16.7%, 2	37.5%, 9	30.6%, 11
Smoking (%, *n*)	33.3%, 4	50%, 12	44.4%, 16
Follicular disorders (acne, folliculitis, pilonidal disease) (%, *n*)	25%, 3	42%, 10	33.3%, 12
Thyroid disease (%, *n*)	8.3%, 1	0%, 0	2.8%, 1
Psoriasis (%, *n*)	8.3%, 1	0%, 0	2.8%, 1
Pyoderma gangrenosum (%, *n*)	0%, 0	4.2%, 1	2.8%, 1
Polycystic ovarian syndrome (%, *n*)	0%, 0	4.2%, 1	2.8%, 1
Depression/anxiety (%, *n*)	0%, 0	4.2%, 1	2.8%, 1
Hypertension (%, *n*)	8.33%, 1	0%, 0	2.8%, 1

*HS*, Hidradenitis suppurativa.

Overall, ectopic HS was seen more commonly in males (67%, *n* = 24), had a late age of onset (mean 40.5 years), and was most commonly reported to present on the head and neck (44.4%, *n* = 16 sites). After stratifying the reported cases of ectopic HS, there were 12 cases of de novo ectopic HS (13 anatomical sites) and 24 cases of classic ectopic HS (34 anatomical sites) (Table I). The percentage of males (67%) and females (33%) presented equally in both groups. The average age of presentation for the cases of de novo and classic ectopic HS was 44.6 years and 38.5 years, respectively.

We found the most common anatomical sites affected were on the trunk (33.3%, *n* = 4) and extremities (33.3%, *n* = 4) for cases of de novo ectopic HS and on the head and neck (54.2%, *n* = 13) for classic ectopic HS. The most common reported comorbidities in cases of de novo ectopic HS were diabetes (42%, *n* = 5), followed by smoking (33.3%, *n* = 4). In cases of classic ectopic HS, the most common comorbidities reported were smoking (50%, *n* = 12), followed by follicular disorders (42%, *n* = 10). Surprisingly, only 8.3% (*n* = 2) of patients with classic ectopic HS had a reported history of diabetes.

Understanding ectopic HS and how it can present differently in patients, in its de novo form or in the setting of classic HS, is important to not only understand and recognize the different presentations of HS that may go unrecognized or misdiagnosed but also to understand the risk factors and patient characteristics that may be associated with these clinical variants. Review of the cases of ectopic HS in the literature found that patients with ectopic HS were more commonly male and presented with these types of lesions later in life. This is different from the classic description of HS, which is typically reported to be more common in females and presents often around the second decade of life.^30^^,^^31^

We stratified the reported ectopic cases by de novo and classic presentations because we believe these may represent different clinical variants, as often de novo cases of ectopic HS are much more difficult to diagnose. In our descriptive analysis, we found differences within these two groups of patients. Notably, patients with de novo ectopic HS had lesions more commonly on the trunk and extremities and had higher rates of diabetes compared to the classic ectopic HS group (42% vs 8.3%, respectively). Meta-analysis studies have shown that the proportion of HS patients with concomitant type 2 diabetes is approximately 16.1%.^32^ Given that we observed higher rates of diabetes in patients with de novo ectopic HS, it may be possible this condition could be a triggering factor in genetically susceptible individuals. This suggests that closer monitoring for diabetes in these patients may be advisable.

Although rates of smoking were high in both groups of patients, they were higher in the classic ectopic HS group, in which half of reported cases listed smoking as a comorbidity. It is known that smoking is likely an exacerbating factor for patients with HS, but this finding suggests it is also possible that those patients with HS who smoke may be at higher risk for developing ectopic HS lesions. In addition, those patients with classic ectopic HS had higher rates of other follicular disorders (acne, folliculitis, pilonidal disease) compared to those patients with de novo ectopic HS. This may indicate that those patients with classic ectopic HS, who also have classic HS lesions, are more susceptible to follicular occlusion compared to those with de novo ectopic HS, possibly reflecting distinct genetic signatures that may be driving these different presentations.

Regardless of prior HS history status, the ectopic lesions seen across all reported cases, including the 2 cases we presented, appear to be secondary to considerable mechanical stress (eg, friction, pressure, shear forces, etc.) and/or trauma (eg, cesarean, amputation, hernia repair, etc.), consistent with the Koebner phenomenon, which is well reported in the HS literature.^4^ The inflammatory milieu and genetic susceptibility of a given patient may certainly predispose to the development of HS lesions in areas of trauma. However, a definitive mechanism accounting for why localized ectopic lesions occur in patients that are lacking a classic HS presentation warrants further investigation. Given the fact that all descriptions of ectopic HS appear to be at sites of friction or trauma, the term “ectopic HS” should be reexamined. In addition, the existing literature describing various HS phenotypes should likely be adapted to include these 2 distinct presentations of ectopic HS.^33^

The clinical challenge of patients presenting with ectopic HS, especially de novo ectopic HS, is recognizing the diagnosis itself. Delays in proper treatment may occur in patients with de novo ectopic HS, given that these solitary lesions may be misdiagnosed as infectious abscesses, inflamed cysts, carbuncles, or other conditions that may mimic HS. The treatment approach for ectopic HS does not differ from the treatment of HS and may include the utilization of topicals, oral antibiotics, biologics such as adalimumab or secukinumab, laser, and surgical treatments such as deroofing or excision. In the patient cases we presented, for example, clinical improvement was seen in one patient with the initiation of adalimumab while the other patient responded to surgical deroofing.

This retrospective review has its limitations. The number of cases published in the current literature is limited, leading to a small sample size, which only allows for descriptive analysis and not true statistical analysis. The number of de novo cases reported in the literature may be underrepresented given that this entity is difficult to recognize clinically. In addition, the retrospective nature of the review limits the information that can be gathered for each case; for example, demographics, comorbidities, history, and clinical descriptions reported in these cases were not standardized, making it difficult to accurately report findings and trends.

Across the literature, HS has earned its place in the realm of complex skin disease with many phenotypic variants described. We believe de novo ectopic HS and classic ectopic HS are likely clinically distinct variants of HS. As noted in the cases of de novo ectopic HS we presented, diagnosis may be difficult to recognize, and as our review of the literature found, it is possible that this clinical variant may be associated with different risk factors compared to the classic presentation. Further research into ectopic HS should be done to continue to investigate the differences in these clinical variants.

## Conflicts of interest

None disclosed.
